# Detection and Characterization of Subvisible Aggregates of Monoclonal IgG in Serum

**DOI:** 10.1007/s11095-012-0749-x

**Published:** 2012-03-31

**Authors:** Vasco Filipe, Robert Poole, Olubukayo Oladunjoye, Kevin Braeckmans, Wim Jiskoot

**Affiliations:** 1 Division of Drug Delivery Technology Leiden/Amsterdam Center for Drug Research, Leiden University, P.O. Box 9502, 2300 RA Leiden, The Netherlands; 2 Department of Pharmaceutics Utrecht Institute for Pharmaceutical Sciences (UIPS), Utrecht University, P.O. Box 80082, 3508 TB Utrecht, The Netherlands; 3 Biophotonic Imaging Group Laboratory of General Biochemistry & Physical Pharmacy Faculty of Pharmacy, Ghent University, Harelbekestraat 72, 9000 Ghent, Belgium; 4 Centre for Nano- and Biophotonics, Harelbekestraat 72, 9000 Ghent, Belgium

**Keywords:** confocal laser scanning microscopy, flow cytometry, monoclonal antibody, serum, subvisible protein aggregates, fluorescence single particle tracking

## Abstract

**Purpose:**

To detect and characterize the aggregation of therapeutic monoclonal antibodies in undiluted biological fluids.

**Methods:**

Fluorescently labeled subvisible IgG aggregates formed by applying either heat stress or by pH-shift were investigated immediately after addition to human serum, and after 24 h. Unstressed and stressed IgG formulations were analyzed by fluorescence single particle tracking, confocal laser scanning microscopy and flow cytometry.

**Results:**

Unstressed formulations remained free from subvisible aggregates in serum, whereas heat-stressed and pH-shift stressed formulations showed dissimilar aggregation behaviors. The aggregation profile of the heat-stressed formulation diluted in serum remained practically the same as the one diluted in buffer, even after the 24 h incubation period. The pH-shift stressed formulation had strikingly smaller and more numerous subvisible aggregates immediately after dilution in serum compared to buffer. These aggregates became noticeably larger in both diluents after 24 h, but in serum they appeared to be formed by other types of constituents than the labeled protein itself.

**Conclusion:**

These results show that subvisible therapeutic protein aggregates may undergo changes in number, type and size distribution upon contact with human serum. This emphasizes the importance of analytical strategies for monitoring aggregation in undiluted biological fluids.

**Electronic supplementary material:**

The online version of this article (doi:10.1007/s11095-012-0749-x) contains supplementary material, which is available to authorized users.

## INTRODUCTION

Therapeutic proteins are an increasingly important class of drugs. However, their inherent tendency to aggregate during manufacture, shipping, storage and delivery remains a problem that hinders their development and commercialization (1–4). The presence of aggregates in protein formulations is undesirable, not only because of reduced therapeutic efficacy due to loss of the active (usually monomeric) form of the protein, but also because it is believed that aggregates can trigger unwanted immunological responses once administered to patients (5–7). The development of an immune response against a therapeutic protein can have serious clinical consequences, such as loss of therapeutic efficacy or even the neutralization of an equivalent endogenous protein (6). Monitoring the amount and type of aggregates present in protein formulations has become a main concern for pharmaceutical companies and regulatory agencies over the last few decades.

Among the numerous types of protein aggregates, subvisible aggregates have received a lot of attention recently because of their potential immunogenicity in conjunction with the fact that they have been analytically overlooked until recent years (3,8). Subvisible aggregates are typically between 0.1 and 50 μm in size and in the present work they are divided into micron- (1–50 μm) and submicron-sized (0.1–1 μm) aggregates. Such aggregates are a particular cause for concern because they mimic highly immunogenic viruses and bacteria both in terms of size range and in terms of the presence of closely spaced repetitive epitopes at their surface (9–11). However, there remain no regulations against the presence of subvisible particles under the size of 10 μm in protein formulations and other parenteral solutions.

The characterization of protein aggregates is complex and requires the use of many different analytical techniques (3,4,12). Until only a few years ago, subvisible aggregates posed a particular analytical challenge, mostly due to the lack of suitable techniques for their size range. This is now changing with the continuing development of new analytical techniques such as nanoparticle tracking analysis (NTA), flow microscopy and Taylor dispersion analysis (TDA) (13–17). However, methods to investigate subvisible aggregates in serum are still lacking and very little is known about the fate of protein pharmaceuticals and their aggregates following administration to patients. The size and affinity of complexes between a therapeutic IgG and its antigen have shown to be different in buffer and serum (18), which reinforces the importance of studying therapeutic protein aggregates also in biological fluids.

The main obstacle that must be overcome in order to analyze aggregates of a specific therapeutic protein in biological fluids is that such fluids contain an extremely high amount of various proteins and other biological components, which have a camouflaging effect for most conventional analytical techniques. To overcome this problem we covalently labeled our protein of interest (IgG) to a fluorescent probe (Alexa Fluor® 488) in order to make it distinguishable from all the other biological components. A wide size range of protein aggregates was obtained by the individual manipulation of two pharmaceutically relevant stress factors: temperature and pH. Stressed and unstressed formulations were then introduced in undiluted human serum and subvisible aggregates were analyzed by three different fluorescence-based techniques: fluorescence single particle tracking (fSPT), confocal laser scanning microscopy (CLSM) and flow cytometry (FCM).

fSPT is an emerging technique for sizing fluorescent particles from about 50 nm to 1 μm. This technique combines a fluorescence microscope with widefield laser illumination and an electron-multiplying charge-coupled device camera, which enables the visualization, recording and size measurement of fluorescent particles moving under Brownian motion. fSPT has already proven to be efficient to size submicron particles in undiluted biological fluids (19,20).

CLSM is a powerful imaging technique that manages to eliminate out-of-focus light in areas that are thicker than the focal plane (ca. 0.75 μm). In this work we decided to use CLSM to monitor the aggregates in serum because this technique removes a great percentage of the background light coming from A488-IgG monomers, which enabled us to have clear images of the aggregates.

FCM is a well-established technique that has the capacity of simultaneously analyzing different parameters of individual particles at a very fast rate, ignoring all other particles that do not meet certain chosen criteria, i.e. serum components in our case. Even though FCM is most commonly used for cells and particles in the micron range, the potential of this technique for analyzing submicron particles is well-known and it has been explored to analyze nanoparticles in biological fluids (21–23).

This work shows that it is possible to answer some of the questions about the fate of biopharmaceuticals upon administration to patients. Monitoring the fate of therapeutic proteins and their aggregates in biological media may provide crucial information for drug development at various phases and help to understand some of the adverse reactions observed for some these drugs.

## MATERIALS AND METHODS

### IgG and Diluents

A recombinant human monoclonal antibody of the IgG1 subclass was used for this experiment at an initial concentration of 1 mg/ml. The buffer used to formulate and dilute the IgG contained 10 mM sodium citrate (Merck, Darmstadt, Germany), 5 % (w/v) sucrose (Sigma-Aldrich, Buchs, Switzerland), pH 6.0. The buffer was filtered using a 0.22-μm PES low binding syringe-driven filter unit (Millex*™* GP, Millipore, Ireland).

Human serum was collected from four healthy volunteers free of medications. The serum was collected in Vacutainer SST tubes (Becton Dickinson, Franklin Lakes, NJ, USA) and centrifuged for 15 min at 3000 rpm in a Beckman Coulter Alegra X-12 centrifuge (Brea, CA, USA) to remove all the blood cells and clotting factors. The serum samples were stored at 4 °C for a maximum period of six hours before being used for the experiment. The viscosity of the buffer and serum was measured in an AR-G2 rheometer from TA Instruments (New Castle, DE, USA) at 37 °C. The average values obtained for buffer were 0.8 cP and for the four collected sera were 1.23, 1.29, 1.30 and 1.34 cP. These values were used for fSPT measurements, in order to obtain accurate size distributions in each diluent. The results in serum displayed in fSPT, CLSM and FCM graphs were chosen from a representative donor in each case.

### Fluorescent Labeling

Alexa Fluor 488 carboxylic acid, N-hydroxysuccinimide ester was obtained from Invitrogen (Merelbeke, Belgium). The IgG labeling was performed according to the manufacturer’s instructions, using an IgG concentration of 10 mg/ml and a molar ratio of 4:1 (dye:IgG). A pH of 7.4 was chosen for the labeling buffer, in order to achieve selective labeling of the amine termini. The A488-IgG was dialyzed using a Float-A-Lyzer® G2 (Spectrum, Rancho Dominguez, CA, USA) with a 100 kDa molecular weight cut-off membrane to remove excess of dye and to exchange from the labeling buffer back to the original formulation buffer. The final A488-IgG concentration was 1 mg/ml and the labeling ratio achieved was about 2 A488 labels per IgG.

### Preparation of IgG Aggregates

The IgG aggregates were obtained by either heat or pH-shift stress. Both labeled and unlabeled IgGs were stressed at a concentration of 1 mg/ml. The heat stress consisted of incubating the A488-IgG at 74 °C for 12 min and the unlabeled IgG at 74 °C for 18 min. One ml of IgG formulation was placed in 1.5-ml reaction tubes (Eppendorf, Hamburg, Germany) and the incubation was performed on an Eppendorf Thermomixer® R (Hamburg, Germany). The pH-shift stress consisted of changing 5 times the buffer pH from pH 6 to pH 1 and back to pH 6 at room temperature. For each pH-shift cycle, hydrochloric acid (5 M) (Sigma-Aldrich, Steinheim, Germany) was slowly added drop wise to the IgG formulation in order to change the pH from 6.0 to 1.0. The samples were then kept for 1 min at this low pH with constant stirring at 400 rpm with a stirring bar. Then, sodium hydroxide (5 M) (Sigma) was added drop wise to adjust the pH back to 6.0. Stirring by itself did not induce aggregation, according to different techniques. All stressed samples were kept at 4 °C until further use. A488-IgG stressed and unstressed formulations were diluted 50-fold in either buffer or serum before fSPT, CLSM and FCM measurements. These samples were analyzed right after the dilution and after an incubation period of 24 h at 37 °C in a INB 400 Memmert incubator (Memmert, Schwabach, Germany).

### Size Exclusion Chromatography (SEC)

SEC was performed on a TSK Gel 3000 SWXL column (Tosoh Bioscience, Montgomeryville, PA, USA), using a Thermo Separation Products Spectra System P4000 gradient pump (Thermo Scientific, Breda, The Netherlands), a Waters 717 plus autosampler (Waters, Milford, MA, USA) and a Spectra-Physics UV150 UV detector (Spectra-Physics, Irvine, CA, USA) at a 280 nm wavelength. The data was collected using ADChrom software version 3.5 (Agilent Technologies, Santa Clara, CA, USA). Fifty μl of each sample was injected and separation was performed at a flow rate of 0.5 ml/min. The running buffer was composed of 25 mM phosphate, 125 mM arginine, 0.025 % (w/v) sodium azide at pH 7 (Sigma-Aldrich, Steinheim, Germany).

### Sodium Dodecyl Sulfate Polyacrylamide Gel Electrophoresis

SDS-PAGE was performed with a Biorad Mini-Protean 3 module (Bio-Rad, Hercules, CA, USA), as described previously (24). Briefly, a 4–20 % linear gradient Tris–HCl Ready Gel from Bio-Rad was run under non-reducing and reducing (sample buffer containing 5 % (v/v) β-mercaptoethanol) conditions at 150 V at room temperature. The bands were detected by Coomassie Brilliant Blue R-250 staining and the gel was scanned with a Bio-Rad GS-800 densitometer and Quantity One software.

### Nanoparticle Tracking Analysis

NTA measurements were performed with a NanoSight LM20 (NanoSight, Amesbury, United Kingdom), equipped with a sample chamber with a 640-nm laser and a Viton fluoroelastomer O-ring, as described previously (15). Briefly, the samples were injected in the sample chamber with sterile BD Discardit II syringes (Becton, Dickinson and Company, Franklin Lakes, NJ, USA) until the liquid reached the tip of the nozzle. The software used for capturing and analyzing the data was the NTA 2.0 Build 127. The samples were measured for 40 s with manual shutter and gain adjustments. Six measurements of each sample were performed and the mean was obtained. The error shadows represent the standard deviations between the measurements. Stressed formulations were diluted 50-fold with buffer before each measurement.

### Light Obscuration (LO)

LO measurements were performed on a PAMAS SVSS system (PAMAS GmbH, Rutesheim, Germany) equipped with a HCB-LD-25/25 sensor and a 1- ml syringe. Each sample was measured three times, with each measurement consisting of a pre-run volume of 0.3 ml followed by three runs of 0.2 ml at a flow rate of 10 ml/min. The final results are a mean of the three runs and the error bars represent the standard deviation between them. Stressed formulations were diluted 50-fold with buffer before each measurement.

### Fluorescence Single Particle Tracking

The fSPT technique was recently described in detail by Braeckmans *et al.* (19). The measurements were performed with an inverted epi-fluorescence microscope (Nikon TE2000E, NIKON BELUX, Brussels, Belgium) equipped with a Nikon Plan Apochromat 100× NA1.4 oil immersion objective lens, a 100 mW Calypso 491 nm laser (Cobolt, Solna, Sweden), an electron-multiplying CCD camera (Cascade II:512; Roper Scientific, Tucson, AZ) and the results were processed with Nikon Elements R imaging software combined with custom build software. Each measurement consisted of the recording and analysis of 10 or 20 videos of the same sample, with the number of videos depending on the amount of aggregates in each sample. Each video was recorded at 35 frames per second for 5 s only in order to minimize photobleaching. At least 3 measurements from each stressed formulation were performed in buffer and 1 measurement for each blood donor.

### Confocal Laser Scanning Microscopy

CLSM images were obtained using the Argon blue laser (488 nm) from a Bio-Rad Radiance 2100 MP confocal laser scanning system (Bio-Rad, Hercules, CA, USA) equipped with a Nikon Eclipse TE2000-U inverted fluorescence microscope and a 60x A/1.4 oil Nikon objective (Nikon, Tokyo, Japan). LaserSharp 2000 v6.0 software (Bio-Rad, Hercules, CA, USA) was used for image acquisition and Adobe Photoshop CS5 (Adobe, San Jose, CA, USA) for image processing. A 4 μl drop of each sample was placed on a well of a 24 well Greiner SensoPlate (Greiner Bio-One, Alphen a/d Rijn, The Netherlands), covered with a glass coverslip (Menzel-Gläser, Braunschweig, Germany) and immediately analyzed. Triplicates of each sample in buffer and serum from each donor were analyzed. The scans were made at a distance of about 10 μm from the glass surface, with the same settings for all submicron aggregates in both diluents. The settings had to be individually adjusted for each micron-sized aggregate in order to obtain sharp images. A rough estimation of the amount of aggregates bigger than ca. 5 μm/ml (i.e. aggregates that had a defined shape) was obtained by extrapolating the average amount of these aggregates present in each well (4 μl). At least 3 wells from each stressed formulation in buffer and serum were considered for these estimations.

### Flow Cytometry

FCM was performed with a BD FACSCanto II flow cytometer (Becton, Dickinson and Company, Franklin Lakes, NJ, USA). The data was collected with the BD FACSDiva 6.2 software and processed with the FlowJo 7.6.4 software (Tree Star, Ashland, OR, USA). The samples were analyzed with the lowest flow rate and the window extension was set to the minimum value (0.5) in order to minimize coincidence of particles in front of the detectors. The measurements were stopped after 2 min of data collection so that the number of events could be compared between samples. At least 3 measurements from each stressed formulation were performed in buffer and 1 measurement for each blood donor. The events were detected by the 530/30 (FITC) and 488/10 (side scatter) photomultiplier tubes (PMTs). Given that most of the aggregates present in our stressed formulations were in the submicron range, the PMT voltages were adjusted for this size range. A threshold of 300 was set for the FITC-PMT in order to eliminate background events from serum.

Yellow-green fluorescent (excitation 505 nm/emission 515 nm) polystyrene standards of 200, 500 and 2000 nm (Invitrogen, Merelbeke, Belgium) were used to optimize PMT voltages for submicron particles. These beads were also used to obtain an approximate size calibration for the protein aggregates, by direct comparison of side scatter intensity and by pulse width calibration (Supplementary Material) (22,25). These two approaches gave similar aggregate size averages.

## RESULTS

A first step in any study involving fluorescently labeled proteins is to identify if and how the presence of the fluorescent probe affects the behavior of the protein. In this case the effect on the aggregation behavior was evaluated.

### Labeling Effect on Protein Aggregation

**Fig. 1:**
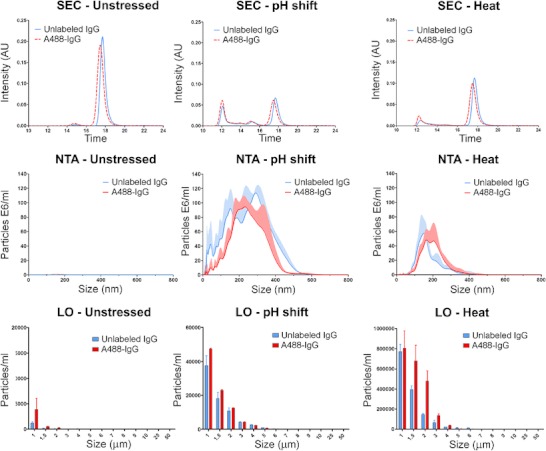
Aggregation profile of A488-IgG and unlabeled IgG formulations, after pH-shift and heat stresses. The top row graphs are chromatograms obtained by SEC with UV absorption detection at 280 nm. The middle row graphs show the aggregate size distribution obtained by NTA (submicron range) and the lower row graphs the distribution obtained by LO (micron range). The NTA and LO graphs contain standard deviations, represented by shadows and error bars, respectively.

In this study Alexa Fluor® 488 (A488) was covalently linked to a humanized monoclonal antibody of the IgG1 subclass (IgG). Incubation at a high temperature (heat stress) and short exposures to low pH (pH-shift stress) were the two stress methods used to obtain subvisible aggregates of both A488-IgG and unlabeled IgG. The effect that this fluorescent label had on the aggregation profile of this protein was monitored by complementary methods covering a broad size range: SEC, NTA and LO (Fig. 1).

These results show very similar aggregation profiles between the A488-IgG and unlabeled IgG, for both stressed and unstressed formulations. SEC results show approximately the same amount of monomer loss and the same pattern of aggregation after the stresses. The 0.2 min left-shift observed in all the SEC chromatograms of the A488-IgG is most likely associated with the slightly increased molecular weight caused by the fluorescent label. NTA and LO results show small differences in size distribution and particle counts between the A488-IgG and the unlabeled IgG aggregates, but they are within the errors associated with the stress methods and analytical procedure.

### Aggregation Profile Characterization

The SEC chromatograms in Fig. 1 show that pH-shift stress induced a large monomer loss associated with the formation of dimers, trimers and larger oligomers. Heat stress led to a slightly smaller monomer loss and the formation of larger oligomers. It is important to notice that with SEC, aggregates larger than 500 kDa either pass through the void volume of the column and appear as a single peak (eluting at ca. 12 min) or they become trapped in the column due to their large size or non-specific binding (26). In fact the protein recovery of the stressed samples was about 70 % and 50 % for the pH-shift and the heat stress samples, respectively. In order to obtain the size distribution of the larger oligomers, NTA and LO were used.

From the NTA results, it is clear that pH-shift stress induced a slightly higher amount and a more polydisperse distribution of submicron aggregates than heat stress. The NTA size averages of the pH-shift and heat-stressed formulations were about 280 nm and 180 nm, respectively. The LO results show a much higher amount of micron-sized aggregates induced by heat than by pH-shift stress. The main reason for the apparent absence of aggregates between 500 nm and 1 μm, when comparing NTA and LO results, is the 10^2^ fold difference in the concentration range detected by these techniques. This means that most aggregates of these stressed formulations are in the submicron range.

**Fig. 2:**
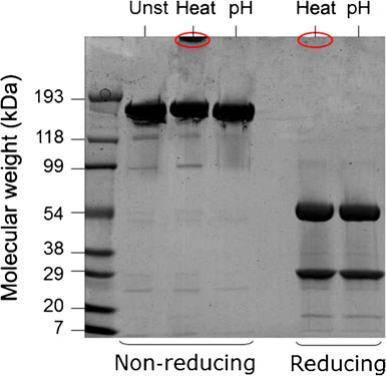
SDS-PAGE gel of unstressed (Unst), heat-stressed (Heat) and pH-shift (pH) stressed formulations under reducing and non-reducing conditions.

Both stressed formulations were analyzed by sodium dodecyl sulfate polyacrylamide gel electrophoresis (SDS-PAGE) in order to detect the presence of covalent aggregates (Fig. 2).

Under non-reducing conditions all the formulations show a pronounced band at around 150 kDa, which corresponds to the molecular weight of IgG monomers. It is also possible to distinguish a clear band at the top of the heat stress lane, which corresponds to covalent aggregates that due to their large size could not enter the gel matrix and therefore deposited on top of the gel. The lack of band at the top of the pH-shift lane suggests that the aggregates formed by this stress method were not covalent and therefore dissociate under the denaturing conditions caused by the presence of SDS.

Under reducing conditions the intramolecular disulfide bonds of the IgG monomers break, originating two bands at around 25 kDa and 50 kDa, which correspond to the light and heavy chains, respectively. Moreover, the absence of a heat-stressed aggregate band (visible on top of the non-reducing gel) under reducing conditions substantiates the covalent, disulfide bond mediated nature of these aggregates.

### Strategy to Monitor Subvisible Aggregates in Serum

In order to monitor the aggregation profile of A488-IgG in serum, unstressed, heat-stressed and pH-shift stressed formulations were diluted at 1:50 (v/v) ratio in buffer and human serum, and analyzed by fSPT, CLSM and FCM. The final IgG concentration after dilution in this experiment (0.02 mg/ml) is in the same order of magnitude as typical concentrations reached by therapeutic IgGs administered intravenously to humans (27,28). The samples were analyzed immediately after dilution and after an incubation period of 24 h at 37 °C.

### Fluorescence Single Particle Tracking

**Fig. 3:**
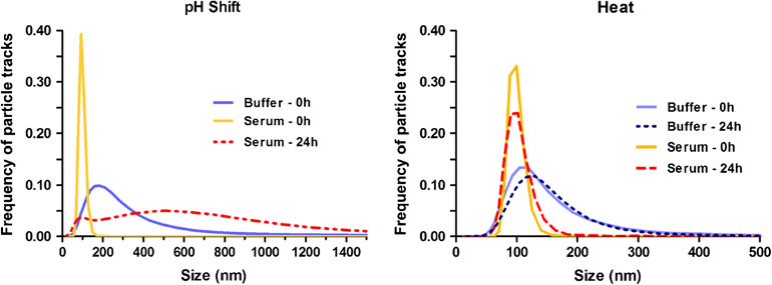
fSPT measurements of stressed A488-IgG formulations in buffer and in serum, immediately after dilution and after a 24 h at 37 °C.

fSPT measurements of A488-IgG stressed formulations in buffer and serum are shown in Fig. 3. The unstressed formulation did not contain measurable amounts of submicron aggregates in either buffer or serum, even after the incubation period at 37 °C.

The fSPT aggregate size distributions in buffer are consistent with the ones obtained by NTA, with size averages for the pH-shift and heat-stressed formulations of 292 nm and 137 nm, respectively (Table I). The differences in size distribution smoothness given by these techniques are mostly due to their different approaches of data handling, as described previously (15,19).

**Table I:** fSPT Mean Size Distribution and Average Aggregate Concentrations from CLSM and FCM Measurements of Stressed A488-IgG Formulations in Buffer and Serum, Immediately After Dilution and After an Incubation Period of 24 h at 37 °C. The Errors Represent the Standard Deviation Between 3 Different Dilutions in Buffer or Between the Measurements in Serum of the Different Blood Donors

Analytical method	Sample	pH-shift	Heat
fSPT mean (nm)	Buffer 0 h	292 ± 47	137 ± 38
	Serum 0 h	110 ± 32	102 ± 27
	Buffer 24 h	N/A	150 ± 46
	Serum 24 h	463 ± 81	112 ± 41
CLSM [(particles > 5 μm) × 10^3^/ml]	Buffer 0 h	10 ± 2	33 ± 7
	Serum 0 h	12 ± 3	40 ± 5
	Buffer 24 h	9 ± 3	35 ± 4
	Serum 24 h	11 ± 3	35 ± 7
FCM (counts × 10^3^)	Buffer 0 h	12 ± 2	11 ± 2
	Serum 0 h	4 ± 1	6 ± 2
	Buffer 24 h	30 ± 8	10 ± 3
	Serum 24 h	90 ± 42	5 ± 2

The size average of the aggregates in the pH-shifted formulation considerably decreased after dilution in serum, from 292 nm to 110 nm. In fact, the amount of aggregates observable through the microscope of this device was strikingly higher in serum right after dilution, but most of them were actually too small and faint for the software to track them properly. This suggests that most of these aggregates were smaller than 50 nm and/or composed of a mixture of A488-IgG and serum components, which would have resulted in aggregates with lower fluorescence intensity. Surprisingly, after the incubation period of 24 h at 37 °C most aggregates in buffer were micron-sized, i.e. beyond the range of fSPT to determine their size. In serum, most of the pH-shift aggregates also became bigger (ca. 460 nm), but not as much as in buffer.

In contrast, the heat-stressed aggregates showed only a small size average decrease when diluted in serum, from 137 nm to 102 nm. However, given that the error of these measurements is about 30 nm, the decrement is not noteworthy. These aggregates seemed to be stable after the 24 h incubation period in both buffer and serum.

### Confocal Laser Scanning Microscopy

**Fig. 4:**
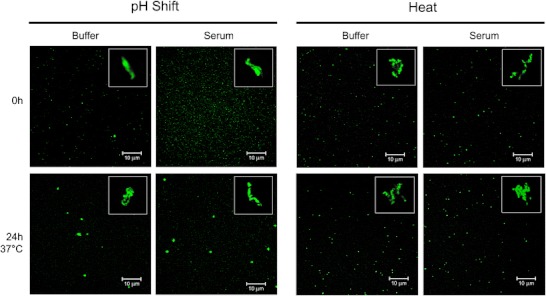
CLSM images of stressed A488-IgG formulations in buffer and serum, immediately after dilution (upper row) and after an incubation period of 24 h at 37 °C (lower row). A representative micron-sized aggregate is shown on the right upper corner of each image (same size scale).

Even though CLSM is primarily used for imaging optical sections of micron-sized structures, the lower size limit of this technique can go beyond the typical optical microscope resolution (ca. 200 nm) if the particles analyzed are bright enough. In this study, while submicron aggregates were captured as mere dots, micron-sized aggregates displayed well-resolved shapes (Fig. 4). This enabled us to also monitor possible morphology and size changes of micron-sized aggregates in serum. It was not possible to distinguish any aggregates for the unstressed formulation in either buffer or serum, even after the incubation period at 37 °C.

The CLSM images show clear differences at time 0 h between the pH-shifted submicron aggregates in buffer and in serum. It is possible to observe that these aggregates became smaller and more numerous immediately after being diluted in serum. After 24 h at 37 °C these aggregates became visibly larger in both diluents, but in this case there were no obvious differences between them. On the other hand, heated submicron aggregates did not seem to change once diluted in serum, even after the incubation period. These observations are consistent with the size distributions obtained by fSPT.

The micron-sized pH-shift aggregates appear to have different morphology from the heat-induced aggregates. pH-shift seems to induce the formation of aggregates resembling clouds or popped balloons, whereas heat-induced aggregates appear to be assemblies of compact smaller units. The morphology and amount (Table I) of these micron-sized aggregates seem to remain approximately the same in buffer and in serum, even after the incubation period.

### Flow Cytometry

**Fig. 5:**
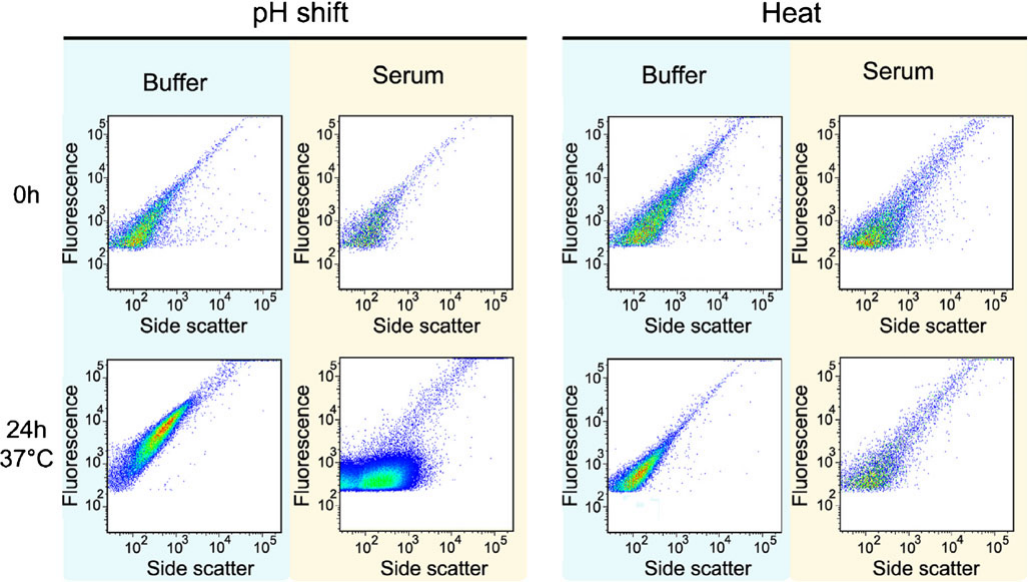
Fluorescence *vs*. side scatter FCM plots of stressed A488-IgG formulations in buffer and serum, immediately after dilution (*upper row*) and after an incubation period of 24 h at 37 °C (*lower row*). The dot density color gradient goes from blue (sparse) to red (very dense).

Fluorescence *vs*. side scatter (SSC) plots of stressed formulations in buffer and serum are shown in Fig. 5. The unstressed formulation gave less than the minimum amount of displayable events for both diluents and both incubation times.

For submicron particles, SSC proved to be the most accurate indicator of particle size, whereas fluorescence intensity served as the selective parameter to distinguish A488-IgG aggregates from other serum components. An approximate calibration with fluorescent standard beads suggested that events with SSC signal of 10^2^ are about 200 nm, 10^3^ are about 500 nm and 10^4^ are micron-sized (Supplementary Material). Although light scatter also depends on the refractive index of the particles, in agreement with fSPT results FCM indicates that most aggregates present in both stressed formulations at time 0 h are about 200 nm. All plots at time 0 h display a linear positive correlation between fluorescence and SSC, indicating that as the size of the aggregates increases, their fluorescence also increases, as expected.

Both stressed formulations show the same dot distribution in serum as in buffer at time 0 h, but the number of events of the pH-shifted and heated formulations in serum is 3 and 2 times lower, respectively (Table I). This occurrence was also observed for fluorescent standard beads, in which the number of events was about 2 times lower in serum than in buffer. Serum seems to have some sort of a masking effect for this technique, most likely caused by its translucent properties. The presence of serum components might lead to secondary scattering and absorption of both the incoming light beam and the scattered or emitted light from the aggregates, eventually resulting in a smaller amount of particle counts.

After the 24 h incubation period, the heat-stressed plot does not suffer any significant changes in both the position of the main population and the number of events (Table I). On the other hand, the main population of the pH-shifted formulation shows a clear shift towards higher values of SSC and fluorescence in buffer after the incubation period, which confirms the formation of larger aggregates. Surprisingly, in serum, the main population shows a shift towards higher SSC, but the fluorescence signal remains the same. The different position of the main population in the 24 h pH-shift plots suggests that the large aggregates formed in serum are somehow different from the ones formed in buffer. Moreover, the number of events after the incubation period increased and it is about 3 times higher in serum than in buffer (Table I).

## DISCUSSION

According to the SDS-PAGE results (Fig. 2), heat stress led to the formation of covalent IgG aggregates, whereas pH-shift stress did not. The nature of these covalent bonds is probably disulfide mediated, as the aggregates were not detected by SDS-PAGE under reducing conditions. Both heat and pH-shift stress conditions are expected to induce at some degree of unfolding, but through distinct driving forces. Protein unfolding normally results in the exposure of hydrophobic regions and eventually, in the case of IgG, free cysteins (29). Protein unfolding is normally followed by aggregation, mostly mediated by hydrophobic effects (30). In the case of heat stress, the formation of intermolecular disulfide bonds is probably facilitated by the high temperature, either by two free cysteins or by thiol-disulfide exchange. Disulfide bonds have smaller dissociation energies than other covalent bonds in the protein and are susceptible to breakage under the reducing conditions used in SDS-PAGE (31). On the other hand, for the pH-shift sample, the low pH prevents the dissociation of thiol groups and consequently the formation of disulfide bonds is hindered (32). This may explain the lack of covalent aggregates at the top of the gel under non-reducing conditions for the pH-shift sample.

The effect that a fluorescent label and a stress factor may have on the aggregation profile of therapeutic proteins, and how this will affect their fate in serum, may vary significantly according to the type of protein, label, stress factor and stress conditions (33–35). Therefore, the results obtained in this study should not be generalized for other proteins or fluorescent labels and a case-by-case approach should be followed.

From all the fluorescent dyes previously tested for this study (Alexa Fluor® 488, 546, 555, 594 and 700), A488 was the one that least changed the aggregation behavior of this IgG for the chosen stress methods (data not shown). However, during optimization studies we noticed that the heat-induced aggregation kinetics of the A488-IgG was slightly different from the unlabeled IgG. At the same temperature, the evolution of the aggregate size distribution was the same, but the A488-IgG had the tendency to arrive faster to these aggregation states. Thus, in order to obtain stressed formulations of labeled IgG with a similar aggregate size distribution as the unlabeled IgG, the heating time of A488-IgG was a few minutes shorter than the one used for unlabeled IgG. Nevertheless, a comparable aggregate size distribution for heated A488-IgG and unlabeled IgG was achieved.

Interestingly, heat-stressed aggregates turned out to be the most measurably stable aggregates in both serum and buffer throughout the incubation period. Therefore, even though the presence of the fluorescent label led to a slightly lower degree of stability of this IgG, this does not seem to have significant consequences for the aggregation state of this protein in serum.

The subvisible aggregates of the pH-shifted formulation were very unstable in serum. The size reduction immediately after dilution in serum was consistently shown by fSPT and CLSM. FCM results did not clearly show this size reduction, most probably because most of them fall under the detection limits of this technique. This size reduction was quite surprising, since in our previous work, in which we tested the potential of fSPT to monitor glutaraldehyde cross-linked covalent aggregates in biological fluids, a slight size increase in serum and plasma was observed, presumably because of adsorption of serum components to these aggregates (20). In the present work, pH-shift aggregates not only became smaller but also much more numerous immediately after dilution in serum. Also the heat-stressed aggregates showed a slight size decrease in serum according to fSPT, although to a lesser extent, which may be due to the partly covalent nature of these aggregates (Fig. 2). These results suggest that something more complex is happening with non-covalent IgG aggregates upon dilution in serum.

The appearance of a very large amount of submicron aggregates in the pH-shifted formulation immediately after dilution in serum was remarkable. Such a high amount suggests that these aggregates derived not only from preexisting aggregates but probably also from unstable monomers. At extreme pH’s proteins are heavily charged, which eventually leads to chemical changes and at least partial unfolding of every monomer in solution (30). When the pH is restored to the original formulation buffer, it is likely that some of these monomers remain partially unfolded. Heat stress also leads to chemical changes and the formation of unfolded states, but by different processes (36). Thus, it is possible that in our case the heat-induced unfolded states were more reversible than the ones caused by pH-shift stress. The presence of unstable pH-shifted monomers and aggregates could then have triggered a multitude of pathways in serum that could have led to the formation of these numerous small submicron aggregates.

The formation of larger subvisible aggregates in both serum and buffer after the incubation period for the pH-shift formulation was also surprising. The aggregation profile of these aggregates had proven to be stable for several weeks, but something as trivial as a dilution with the same buffer induced an aggregate-size increase. This occurrence shows how complex and unpredictable aggregated species can behave. This A488-IgG aggregate size increase upon dilution after 24 h was confirmed with unlabeled IgG and it also happened at 4 °C, but to a lesser extent (data not shown). These results support the concerns about on the one hand analytical techniques for protein aggregates involving dilutions, such as SEC and field flow fractionation, and on the other hand changes that may occur after *e.g.* intravenous administration which is followed by rapid dilution (37).

FCM results suggest that the large pH-shift aggregates formed in serum after the incubation time had a different composition from the ones in buffer. The observation that large aggregates formed in serum were considerably less fluorescent than the ones formed in buffer, led to the hypothesis that the former are composed of a mixture of A488-IgG and other, non-fluorescing serum components. Adsorption of serum proteins to nanoparticles and other biological materials upon dilution in a physiological environment has been reported (38). In fact, pure self-association of A488-IgG molecules in such complex environments is highly unlikely, since specific binding between monomers of the same IgG is not to be expected.

Immunoglobulins are the second most abundant protein in blood and the amount of physiological pathways in which IgG can be involved is enormous (39). Thus, identifying the components responsible for the pH-shift aggregate changes in serum can be very challenging. Nevertheless, we decided to briefly investigate if human serum albumin (HSA) or complement factor C1q could be involved in these changes. We chose these proteins because: HSA is the most abundant protein present in blood and is known for being a carrier for several proteins; C1q is known to bind to IgG only when it is aggregated, in order to activate the classical pathway of the complement system (39,40). The pH-shift formulation was diluted in buffer containing these proteins at their approximate serum concentration (HSA—40 mg/ml; C1q—0.1 mg/ml), but no conclusive results were obtained (data not shown) (39,41). It is likely that a combination of several serum components is required to induce the changes observed in serum. This underscores the need for more research to understand the causes of the observed changes in the subvisible aggregate profile in serum.

The fact that micron-sized aggregates of both stressed formulations did not seem to change throughout the entire experiment is intriguing. Nevertheless, small changes in this size range could have been overlooked by CLSM. Therefore, all samples were also analyzed by FCM with settings optimized for the micrometer range, but no clear differences in particle counts, fluorescence intensity or scattering signal were observed (data not shown). Altogether, it seems that pH-shift induced micron-sized aggregates are more stable than submicron aggregates in serum.

The only condition stipulated by the United States Pharmacopeia (USP) for the presence of subvisible particles in therapeutic protein formulations as well as other parenteral solutions (standard <788>) states that particles >10 μm should be controlled below 6000 particles/container and particles >25 μm below 600 particles/container. However, there are no studies that would support the idea that submicron and micron-sized aggregates are less immunogenic than visible precipitates (>50 μm) (8). In fact, our stressed samples would have passed USP standards and yet most of the product is under the form of potentially immunogenic aggregates, which subsist after dilution in serum.

## CONCLUSIONS

The fate and behavior of subvisible IgG aggregates in biological fluids has been investigated. Whilst unstressed IgG remains apparently unchanged in serum, i.e. it does not spontaneously aggregate, certain aggregates of the same protein do change. Aggregates formed by pH-shift appear particularly susceptible, whereas the ones formed by heat stress seem to be stable. These results indicate that the aggregation profile of therapeutic proteins may drastically change once the formulation is administered, emphasizing the importance of analytical strategies for monitoring aggregation in undiluted biological fluids.

## ELECTRONIC SUPPLEMENTARY MATERIAL

Below is the link to the electronic supplementary material.

ESM 1 (JPEG 205 kb)
