# Supplementary figures and images for: Detection and Characterization of Subvisible Aggregates of Monoclonal IgG in Serum
Source: Pharm Res. 2012 Mar 31;29(8):2202–12. doi: 10.1007/s11095-012-0749-x (PMC3399096; doi:10.1007/s11095-012-0749-x)

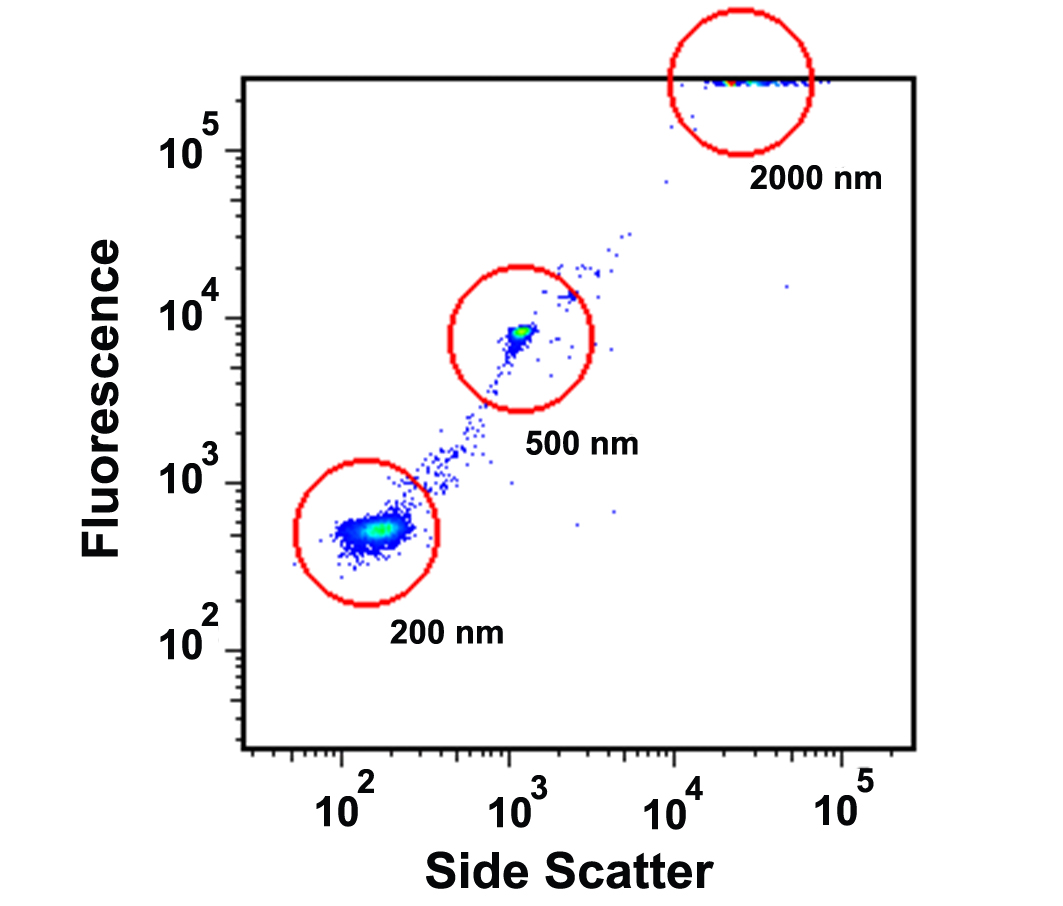

Supplement: Supplementary file 1 — (JPEG 205 kb) [file 11095_2012_749_MOESM1_ESM.jpg]
